# Efficacy and Safety of Antivascular Endothelial Growth Factor (Anti-VEGF) in Treating Neovascular Age-Related Macular Degeneration (AMD): A Systematic Review and Meta-analysis

**DOI:** 10.1155/2022/6004047

**Published:** 2022-04-15

**Authors:** Xiaobei Yin, Ting He, Shanshan Yang, Hui Cui, Wenlan Jiang

**Affiliations:** ^1^Department of Ophthalmology, The Affiliated Hospital of Qingdao University, Qingdao, Shandong 266003, China; ^2^Beijing Puren Hospital, Beijing 100010, China; ^3^Senior Department of Ophthalmology, The Third Medical Center of PLA General Hospital, Beijing 100039, China

## Abstract

This study is aimed at assessing the efficacy and safety of antivascular endothelial growth factor (anti-VEGF) inhibitors in treating age-related macular degeneration (AMD). PubMed, Embase, and Cochrane library were searched. Weighted mean difference (WMD) and relative risk (RR) with 95% confidence interval (CI) were applied to assess outcomes. Eighteen randomized controlled trials involved 8,847 neovascular AMD patients were selected for the meta-analysis. Pegaptanib (WMD: 6.70; *P* < 0.001) and ranibizumab (WMD: 17.80; *P* < 0.001) were associated with greater BCVA changes than control after 1 year. Bevacizumab was linked with less changes in central macular thickness after 1 year compared to control (WMD: -38.50; *P* < 0.001), but more changes compared to ranibizumab (WMD: 10.69; *P* = 0.024). The incidence of gain of 15 or more letter visual acuity after 1 year was increased when compared with bevacizumab versus control (RR: 7.80; *P* = 0.001), pegaptanib versus control (RR: 2.83; *P* = 0.015), and ranibizumab versus control (RR: 3.92; *P* = 0.003). Moreover, ranibizumab was associated with more BCVA changes and an increased incidence of gain of 15 or more letter visual acuity after 2 years compared with control (RR: 5.77; *P* < 0.001). This study found that most anti-VEGF inhibitors provided better efficacy than non-anti-VEGF intervention, and the treatment effectiveness among various anti-VEGF agents was equally effective.

## 1. Introduction

Age-related macular degeneration (AMD) is the leading cause of visual loss in elderly people, and the disease burden of AMD is projected to increase because of ageing populations and rising life expectancies [1–3]. The AMD could divided into neovascular and nonneovascular AMD, and the wet or neovascular AMD contributed an important role for severe visual impairment [4]. The main characteristic of neovascular AMD was choroidal neovascularization involved the growth of abnormal vessels into the retina [5]. Hernández-Zimbrón et al. found that neovascular AMD was associated with increased risk of intraretinal or subretinal leakage, hemorrhage, and retinal pigment epithelium and causing rapid decline in vision [6]. Ocular inflammation is also associated with glial cell proliferation and occlusion of retinal capillaries and vascular changes [7–10]. These results caused high cost and mandatory frequent monitoring visits for patients and healthcare system. Therefore, early detection and effective intervention of advanced neovascular AMD are important for improving visual outcomes [11].

Nowadays, antivascular endothelial growth factor (anti-VEGF) inhibitors were the mainstay treatment strategy for patients with neovascular AMD [3], and studies have already found the use of anti-VEGF inhibitors timely could achieve the treatment goals for improving visual acuity over long periods [12–15]. However, there was concern for long-term that anti-VEGF inhibitors might affect the macula because of VEGF might play an important role on the integrity of the retinal pigment epithelium [16]. Several meta-analyses have already addressed the treatment effectiveness of anti-VEGF inhibitors for neovascular AMD [17, 18]. However, the additional published articles should be entered to update the pooled results because of the efficacy and safety of use long-term anti-VEGF inhibitors were variable. Therefore, we conducted a systematic review and meta-analysis of randomized controlled trials (RCTs) to evaluate the efficacy and safety of anti-VEGF inhibitors for patients with neovascular AMD.

## 2. Materials and Methods

### 2.1. Data Sources, Search Strategy, and Selection Criteria

The Preferred Reporting Items for Systematic Reviews and Meta-Analysis Statement was used to guide the performing and reporting of this study [19]. RCTs investigated that the treatment effectiveness of anti-VEGF inhibitors for neovascular AMD was eligible in our study. The electronic searches were conducted in PubMed, Embase, and the Cochrane library for eligible studies throughout October 2020, and the following search terms were used in text word or Medical Subject Heading: (pegaptanib or ranibizumab or bevacizumab or aflibercept or conbercept) and (neovascular age-related macular degeneration) and (randomized controlled trial). The trial has already completed but not yet published and was also searched in the website ofhttp://clinicaltrials.gov/(US NIH). Moreover, the reference lists of relevant review and original article were manually searched to identify any new eligible trial.

The literature search and study selection were independently performed by 2 reviewers, and any disagreement between reviewers was resolved by group discussion until a consensus was reached. Trial was included if they met as follows: (1) patients: neovascular AMD; (2) intervention and control: pegaptanib, ranibizumab, bevacizumab, aflibercept, conbercept, and non-anti-VEGF inhibitors; (3) outcomes: best corrected visual acuity (BCVA), central macular thickness, gain of 15 or more letter visual acuity, death, and arteriothrombotic events; and (4) study design: the study had to have RCT design.

### 2.2. Data Collection and Quality Assessment

Two reviewers independently abstracted the characteristics of studies and patients and entered into Excel: first author or study group's name, publication year, country, sample size, age, male proportion, size of lesion, total area of choroidal neovascularization, angiographic subtype of lesion, intervention and control, follow-up duration, and reported outcomes. Then, the quality of individual trial was assessed using the Jadad scale by 2 reviewers, which based on the items of randomization, blinding, allocation concealment, withdrawals and dropouts, and use of intention-to-treat analysis [20]. The scoring system of Jadad scale ranged from 0 to 5, and the trial scored 4 or 5 was regarded as high quality. Any inconsistency between 2 reviewers for data collection and quality assessment was settled by an additional reviewer referring to the full text of eligible trials.

### 2.3. Statistical Analysis

The treatment effectiveness of anti-VEGF inhibitors was calculated by weighted mean difference (WMD) and relative risk (RR) with 95% confidence interval (CI) for continuous and categorical outcomes, respectively. In order to account for the wide variety of possible treatment-comparator combinations, the studies that used the same pairs of treatment and comparator were pooled together for the meta-analyses. Then, the random-effect model was used to calculate pooled effect estimates owing to it considering the underlying varies across included trials [21, 22]. Heterogeneity among trials was assessed by using *I*^2^ and *Q* statistic, and significant heterogeneity was defined as *I*^2^ > 50.0% or *P*_*Q* statistic_ < 0.10 [23, 24]. Sensitivity analysis was applied for outcome reported >5 trials to assess the robustness of pooled conclusion by sequential excluding individual trial [25]. Subgroup analyses were also conducted based on intervention, control, and follow-up duration. Publication bias for each outcome was also assessed by using funnel plots, Egger, and Begg tests [26, 27]. The inspection level was 2-sided, and the cutoff value of 0.05 was considered as the treatment effectiveness was associated with statistically significant. All of statistical analyses in this study were conducted using the STATA software (version 10.0; StataCorp, Texas, United States of America).

## 3. Results

### 3.1. Literature Search

**Figure 1:**
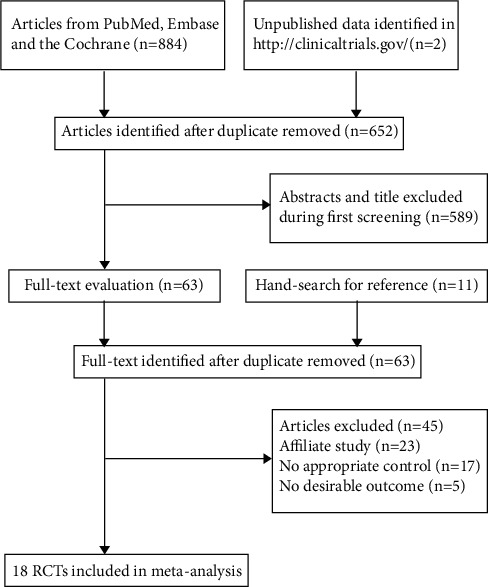
The PRISMA flowchart for the literature search and study selection.

The electronic searches from PubMed, Embase, and the Cochrane library yield 884 articles, and 652 trials were retained after duplicate articles were removed. Additional 589 studies were excluded because of these studies reported irrelevant titles or abstracts. The remaining 63 studies were retrieved for further full-text evaluations, and 45 studies were excluded because of: affiliate study (*n* = 23), no appropriate control (*n* = 17), or did not include the outcome desired for the present meta-analysis (*n* = 5). Reviewing the reference lists of these studies did not found any new eligible trial. After this, a total of 18 RCTs were selected for final meta-analysis [28–45], and the details regarding the study selection process are shown in Figure 1.

### 3.2. Study Characteristics

The included trials published between 2004 and 2019, and the sample size ranged from 22 to 2,412. Sixteen trials were conducted in western countries, and the remaining 2 trials were conducted in eastern countries. The mean age of patients ranged from 63.9 to 80.1 years, and the male proportion ranged from 29.1 to 59.5 percent. Study quality was assessed by Jadad scale, 7 trials scored 5, 8 trials scored 4, and the remaining 3 trials scored 3. The characteristics of included studies are described in more detail in Table 1.

### 3.3. Best Corrected Visual Acuity

**Figure 2:**
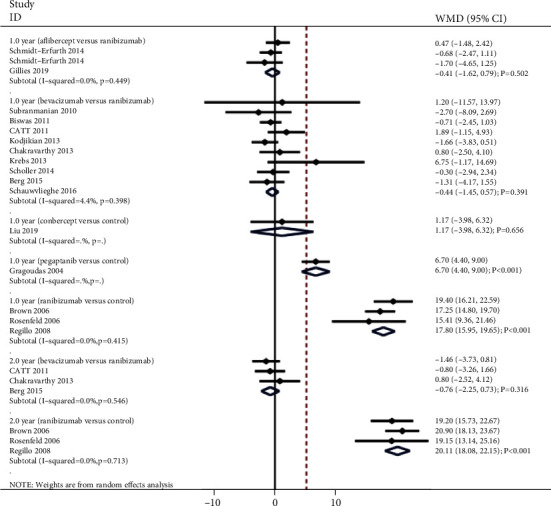
Effect of anti-VEGF inhibitors on the change of BCVA.

The summary results for the effect of anti-VEGF inhibitors on the change of BCVA according to interventions and follow-up are presented in Figure 2. We noted that pegaptanib (WMD: 6.70; 95% CI: 4.40 to 9.00; *P* < 0.001) and ranibizumab (WMD: 17.80; 95% CI: 15.95 to 19.65; *P* < 0.001; no evidence of heterogeneity) were associated with greater changes in BCVA after 1 year when compared with non-anti-VEGF inhibitors. However, conbercept versus control was not associated with the change of BCVA after 1 year (WMD: 1.17; 95% CI: -3.98 to 6.32; *P* = 0.656). Moreover, there were no significant differences for the changes of BCVA after 1 year when comparison aflibercept versus ranibizumab (WMD: -0.41; 95% CI: -1.62 to 0.79; *P* = 0.502; no evidence of heterogeneity) and bevacizumab versus ranibizumab (WMD: -0.44; 95% CI: -1.45 to 0.57; *P* = 0.391; unimportant heterogeneity). Finally, ranibizumab was associated with greater change in BCVA after 2 years when compared with non-anti-VEGF inhibitors (WMD: 20.11; 95% CI: 18.08 to 22.15; *P* < 0.001; no evidence of heterogeneity), while no significant difference between bevacizumab and ranibizumab for the change of BCVA after 2 years (WMD: -0.76; 95% CI: -2.25 to 0.73; *P* = 0.316; no evidence of heterogeneity).

### 3.4. Central Macular Thickness

**Figure 3:**
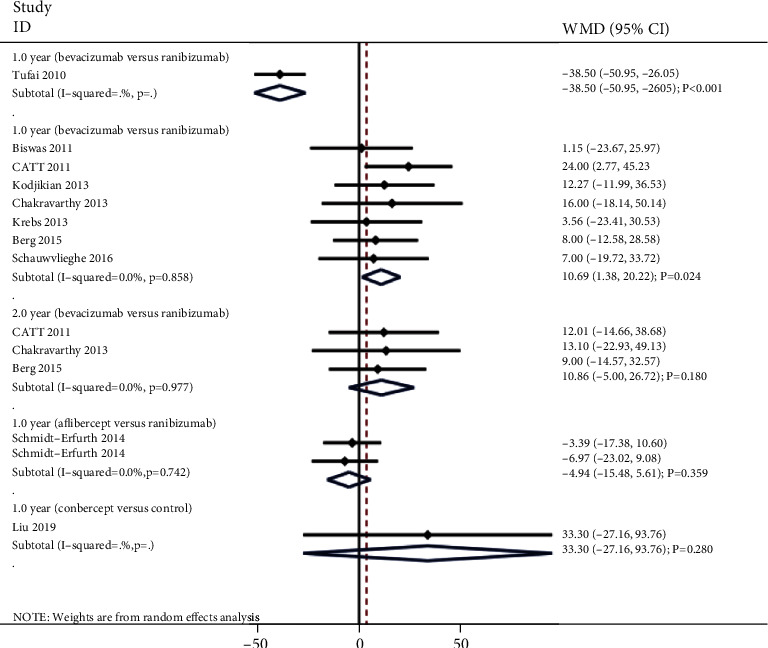
Effect of anti-VEGF inhibitors on the change of central macular thickness.

The summary results for the effect of anti-VEGF inhibitors on the change of central macular thickness according to interventions and follow-up are presented in Figure 3. We noted that bevacizumab versus control was associated with less change in central macular thickness after 1 year (WMD: -38.50; 95% CI: -50.95 to -26.05; *P* < 0.001), while bevacizumab was associated with greater change in central macular thickness after 1 year than ranibizumab (WMD: 10.69; 95% CI: 1.38 to 20.00; *P* = 0.024; no evidence of heterogeneity). However, there were no significant differences for the changes of central macular thickness after 1 year when compared with aflibercept versus ranibizumab (WMD: -4.94; 95% CI: -15.48 to 5.61; *P* = 0.359; no evidence of heterogeneity) and conbercept versus control (WMD: 33.30; 95% CI: -27.16 to 93.76; *P* = 0.280). Finally, bevacizumab was not associated with the change of central macular thickness as compared with ranibizumab (WMD: 10.86; 95% CI: -5.00 to 26.72; *P* = 0.180; no evidence of heterogeneity).

### 3.5. Gain of 15 or More Letter Visual Acuity

**Figure 4:**
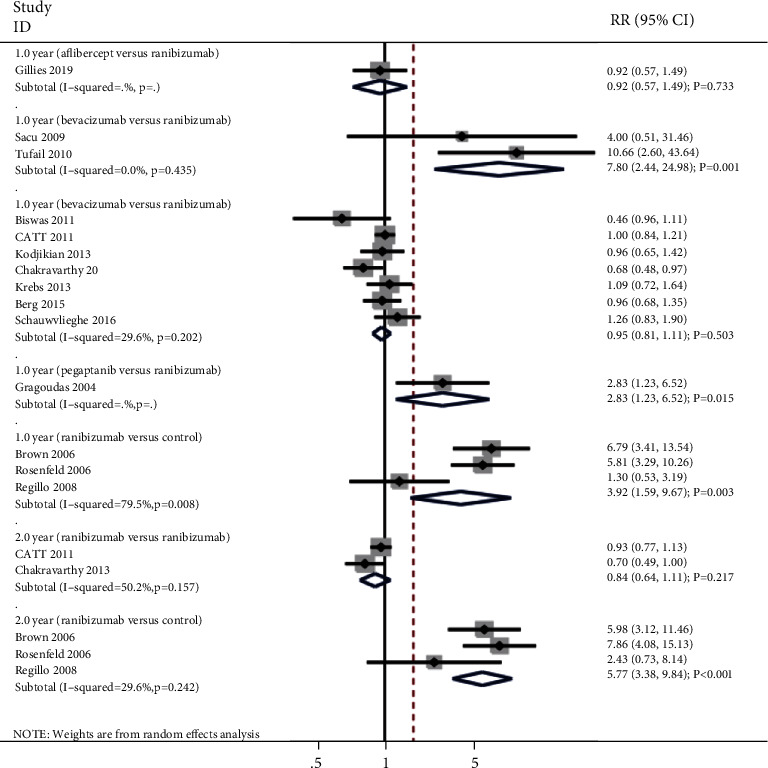
Effect of anti-VEGF inhibitors on the incidence of gain of 15 or more letter visual acuity.

The summary results for the effect of anti-VEGF inhibitors on the incidence of gain of 15 or more letter visual acuity according to interventions and follow-up are presented in Figure 4. We noted that bevacizumab (RR: 7.80; 95% CI: 2.44 to 24.98; *P* = 0.001; no evidence of heterogeneity), pegaptanib (RR: 2.83; 95% CI: 1.23 to 6.52; *P* = 0.015), and ranibizumab (RR: 3.92; 95% CI: 1.59 to 9.67; *P* = 0.003; significant heterogeneity) were associated with an increased incidence of gain of 15 or more letter visual acuity after 1 year when compared with non-anti-VEGF inhibitors. However, there were no significant differences for the incidence of gain of 15 or more letter visual acuity after 1 year when compared with aflibercept versus ranibizumab (RR: 0.92; 95% CI: 0.57 to 1.49; *P* = 0.733) and bevacizumab versus ranibizumab (RR: 0.95; 95% CI: 0.81 to 1.11; *P* = 0.503; unimportant heterogeneity). Finally, we noted that ranibizumab was associated with an increased incidence of gain of 15 or more letter visual acuity after 2 years than non-anti-VEGF inhibitors (RR: 5.77; 95% CI: 3.38 to 9.84; *P* < 0.001; unimportant heterogeneity), while no significant difference between bevacizumab and ranibizumab for the incidence of gain of 15 or more letter visual acuity after 2 years (RR: 0.84; 95% CI: 0.64 to 1.11; *P* = 0.217; significant heterogeneity).

### 3.6. Death

**Figure 5:**
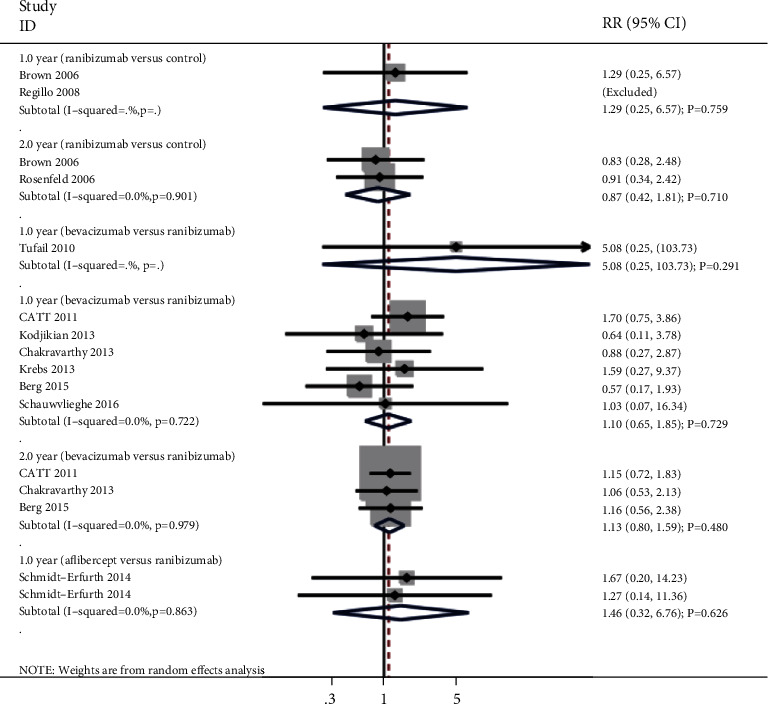
Effect of anti-VEGF inhibitors on the risk of death.

The summary results for the effect of anti-VEGF inhibitors on the risk of death according to interventions and follow-up are presented in Figure 5. There were no significant differences for the risk of death when compared with ranibizumab versus control (RR: 1.29; 95% CI: 0.25 to 6.57; *P* = 0.759), bevacizumab versus control (RR: 5.08; 95% CI: 0.25 to 103.73; *P* = 0.291), bevacizumab versus ranibizumab (RR: 1.10; 95% CI: 0.65 to 1.85; *P* = 0.729; no evidence of heterogeneity), or aflibercept versus ranibizumab after 1 year (RR: 1.46; 95% CI: 0.32 to 6.76; *P* = 0.626; no evidence of heterogeneity), and ranibizumab versus control (RR: 0.87; 95% CI: 0.42 to 1.81; *P* = 0.710; no evidence of heterogeneity), or bevacizumab versus ranibizumab after 2 years (RR: 1.13; 95% CI: 0.80 to 1.59; *P* = 0.480; no evidence of heterogeneity).

### 3.7. Arteriothrombotic Events

**Figure 6:**
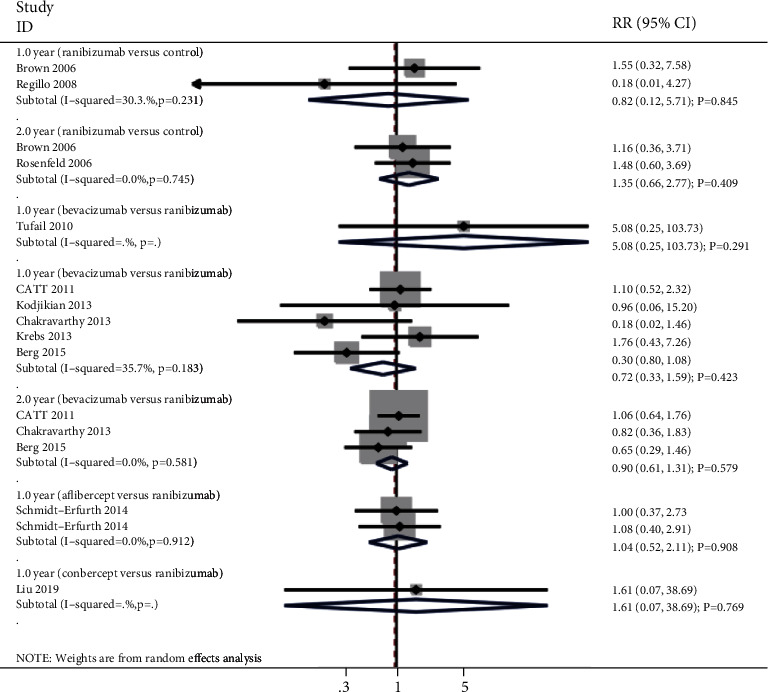
Effect of anti-VEGF inhibitors on the risk of arteriothrombotic events.

The summary results for the effect of anti-VEGF inhibitors on the risk of arteriothrombotic events according to interventions and follow-up are presented in Figure 6. There were no significant differences for the risk of arteriothrombotic events when compared with ranibizumab versus control (RR: 0.82; 95% CI: 0.12 to 5.71; *P* = 0.845; moderate heterogeneity), bevacizumab versus control (RR: 5.08; 95% CI: 0.25 to 103.73; *P* = 0.291), bevacizumab versus ranibizumab (RR: 0.72; 95% CI: 0.33 to 1.59; *P* = 0.423; moderate heterogeneity), aflibercept versus ranibizumab (RR: 1.04; 95% CI: 0.52 to 2.11; *P* = 0.908; no evidence of heterogeneity), or conbercept versus control after 1 year (RR: 1.61; 95% CI: 0.07 to 38.69; *P* = 0.769), and ranibizumab versus control (RR: 1.35; 95% CI: 0.66 to 2.77; *P* = 0.409; no evidence of heterogeneity), or bevacizumab versus ranibizumab after 2 years (RR: 0.90; 95% CI: 0.61 to 1.31; *P* = 0.579; no evidence of heterogeneity).

### 3.8. Publication Bias

Publication bias for each outcome was also assessed and listed in Supplemental 1. We noted potential significant publication bias for BCVA and central macular thickness, while no significant publication bias for gain of 15 or more letter visual acuity, death, and arteriothrombotic events was detected.

## 4. Discussion

The current meta-analysis was systematically assessed the effects of anti-VEGF inhibitors on BCVA, central macular thickness, gain of 15 or more letter visual acuity, death, and arteriothrombotic events for patients with neovascular AMD based on published RCTs. A total of 8,847 neovascular AMD patients from 18 RCTs were included in this study across wide range of patients' characteristics. The results suggested that mostly anti-VEGF inhibitors could yield superior effects on the changes in BCVA (in letters), or central macular thickness, and increased the incidence of gain of 15 or more letter visual acuity. Moreover, the use of anti-VEGF inhibitors did not cause additional risk of death and arteriothrombotic events. These results indicated that anti-VEGF inhibitors could provide better effectiveness and well tolerate for patients with neovascular AMD, which should recommend in clinical practice.

Several meta-analyses have already investigated the treatment effectiveness of anti-VEGF inhibitors for patients with neovascular AMD. A Cochrane review identified 16 RCTs and found that the use of anti-VEGF inhibitors provides better effects on visual acuity, and the difference among various anti-VEGF inhibitors was not associated with statistically significant. Moreover, the use of anti-VEGF inhibitors did not yield additional risk of serious complications [17]. Nguyen et al. conducted a meta-analysis of 15 RCTs and found that bevacizumab and ranibizumab provide equivalent efficacy for BCVA, while ranibizumab was associated with greater reduction in central macular thickness and lower risk of serious systemic complications. Moreover, there were no significant differences between aflibercept and ranibizumab for the changes of BCVA and central macular thickness [18]. However, the pooled results for the use of anti-VEGF inhibitors after 2 years follow-up were variable. Additional published RCTs should be entered into meta-analysis, and the results needed reevaluated. Therefore, the current updated meta-analysis was conducted to systematically assess the effects of anti-VEGF inhibitors on BCVA, central macular thickness, gain of 15 or more letter visual acuity, death, and arteriothrombotic events for patients with neovascular AMD.

This study found that pegaptanib and ranibizumab yield significant effect on the change of BCVA after 1 year, and this effect was persistent for the use of ranibizumab after 2 years. The effect of pegaptanib on the change of BCVA after 1 year was based on 1 trial, and the result might variable [28]. Moreover, bevacizumab was associated with less change in central macular thickness after 1 year. Furthermore, the use of bevacizumab, pegaptanib, and ranibizumab was associated with an increased incidence of gain of 15 or more letter visual acuity after 1 year, and the effect of ranibizumab was persisted on the incidence of gain of 15 or more letter visual acuity after 2 years. Some reasons could explain the above results: ocular VEGF level is synchronous rise with the growth and leakage of new vessels [46–48]. Moreover, the neovascularization in corneal, iridic, retinal, and choroidal from animal models suggested that neovascularization was dependent on the presence of VEGF [49–51]. Furthermore, a recapitulation of the pathologic neovascularization was found when introduction of VEGF into normal animal eyes [52, 53]. Interesting, although no significant difference among various anti-VEGF inhibitors for the changes of BCVA, we noted that bevacizumab produces more change in central macular thickness after 1 year as compared with ranibizumab. This result suggested that mostly anti-VEGF inhibitors yield similar efficacious on the change of BCVA, while ranibizumab could reduce the abnormally increased in central retinal thickness and provide better anatomical outcome [54].

Our study did not found any significant differences for the risk of death and arteriothrombotic events, irrespective comparisons of anti-VEGF inhibitors versus control, or various anti-VEGF inhibitors. These results were consistent with the results from the Nguyen et al.'s study [18]. However, they point out that the use of bevacizumab was associated with an increased risk of at least 1 serious systemic adverse event as compared with ranibizumab, irrespective after 1 and 2 years. The potential reason for these venous thrombotic adverse events was more frequent when treated with bevacizumab.

Although this study provides comprehensive quantitative results, several shortcoming of this study should be mentioned: (1) smaller number of trials reported the effects of anti-VEGF inhibitors after 2 years, and the pooled effect estimates were not robustness; (2) stratified analyses according to the severity of neovascular AMD were not conducted because of this information was not reported in most studies; (3) the analysis of this study used pooled data, and the detail analyses were restricted; and (4) the results of this study are based on published RCTs, and publication bias was inevitable.

## 5. Conclusion

This study found that the use of anti-VEGF inhibitors could yield better efficacious than non-anti-VEGF intervention on BCVA (in letters), or central macular thickness, and increased the incidence of gain of 15 or more letter visual acuity. Moreover, there were no significant differences for the risk of death and arteriothrombotic events among anti-VEGF inhibitors and control intervention. Further large-scale RCT should be conducted to assess the long-term effects of anti-VEGF inhibitors for patients with neovascular AMD.

## Figures and Tables

**Table 1:** The baseline characteristics of identified studies and enrolled patients.

Study	Country	Sample size	Age (years)	Male (%)	Size of lesion	Total area of CNV	Angiographic subtype of lesion	Intervention	Follow-up (years)	Study quality
Gragoudas, 2004 [29]	US, Canada, Europe, Israel, Australia, and South America	1,208	75.5	40.9	4.0	NA	Predominantly classic: 306; minimally classic: 426; occult with no classic: 458	Pegaptanib; sham injection	1.0	5
Brown, 2006 [30]	US, France, Germany, Hungary, Czech Republic, and Australia	423	77.0	50.1	1.9	1.4	Predominantly classic: 410; minimally classic: 12; occult with no classic: 1	Ranibizumab; verteporfin	2.0	5
Rosenfeld, 2006 [31]	US	716	77.0	35.2	4.4	4.2	Predominantly classic: 1; minimally classic: 264; occult with no classic: 451	Ranibizumab; sham injection	2.0	5
Regillo, 2008 [32]	US	184	78.4	40.2	4.2	3.6	Predominantly classic: 35; minimally classic: 69; occult with no classic: 79	Ranibizumab; sham injection	2.0	4
Sacu, 2009 [33]	Austria	28	78.0	32.1	NA	NA	NA	Bevacizumab; triamcinolone	1.0	4
Tufail, 2010 [34]	UK	131	80.0	59.5	6.1	3.5	Predominantly classic: 49; minimally classic: 151	Bevacizumab; verteporfin	1.0	5
Subranmanian, 2010 [35]	US	22	78.6	95.5	NA	NA	Predominantly classic: 3; minimally classic: 4; occult with no classic: 15	Bevacizumab; ranibizumab	1.0	3
Biswas, 2011 [36]	India	104	63.9	48.1	NA	NA	NA	Bevacizumab; ranibizumab	1.5	3
CATT, 2011 [37]	US	1,185	79.3	38.2	NA	NA	NA	Bevacizumab; ranibizumab	2.0	4
Kodjikian, 2013 [38]	France	374	79.7	33.7	NA	1.9	NA	Bevacizumab; ranibizumab	1.0	4
Chakravarthy, 2013 [39]	UK	525	77.7	40.0	3.6	NA	NA	Bevacizumab; ranibizumab	2.0	5
Krebs, 2013 [40]	Austria	317	77.2	36.3	NA	NA	NA	Bevacizumab; ranibizumab	1.0	5
Scholler, 2014 [41]	Austria	55	80.1	29.1	1.9	NA	NA	Bevacizumab; ranibizumab	1.0	3
Schmidt-Erfurth, 2014 [42]	US, Canada, Europe, the Middle East, the Asia-Pacific region, and Latin America	2,412	75.9	42.9	7.6	7.2	Predominantly classic: 631; minimally classic: 838; occult with no classic: 926	Aflibercept; ranibizumab	2.0	4
Berg, 2015 [43]	Norway	431	78.3	32.5	7.0	NA	NA	Bevacizumab; ranibizumab	2.0	5
Schauwvlieghe, 2016 [44]	Netherlands	327	78.0	44.0	2.7	NA	Predominantly classic: 85; minimally classic: 51; occult with no classic: 177	Bevacizumab; ranibizumab	1.0	4
Liu, 2019 [45]	China	124	66.1	67.7	NA	NA	Predominantly classic: 61; minimally classic: 29; occult with no classic: 31	Conbercept; sham injection	1.0	4
Gillies, 2019 [46]	Australia	281	77.6	47.3	NA	NA	NA	Aflibercept; ranibizumab	1.0	4

^∗^CNV: choroidal neovascularization; NA: not available.

## Data Availability

All data generated or analysed during this study are included in this published article and its supplementary information files.

## Supplementary Materials

Supplementary Materials Figure S1: funnel plot for BCVA. Figure S2: funnel plot for central macular thickness. Figure S3: funnel plot for gain of 15 or more letter visual acuity. Figure S4: funnel plot for death. Figure S5: funnel plot for arteriothrombotic events.
