# Supplementary material for: Efficacy and Safety of Antivascular Endothelial Growth Factor (Anti-VEGF) in Treating Neovascular Age-Related Macular Degeneration (AMD): A Systematic Review and Meta-analysis
Source: J Immunol Res. 2022 Apr 15;2022:6004047. doi: 10.1155/2022/6004047 (PMC9033403; doi:10.1155/2022/6004047)
Supplement: Supplementary Materials — Figure S1: funnel plot for BCVA. Figure S2: funnel plot for central macular thickness. Figure S3: funnel plot for gain of 15 or more letter visual acuity. Figure S4: funnel plot for death. Figure S5: funnel plot for arteriothrombotic events. [file 6004047.f1.docx]

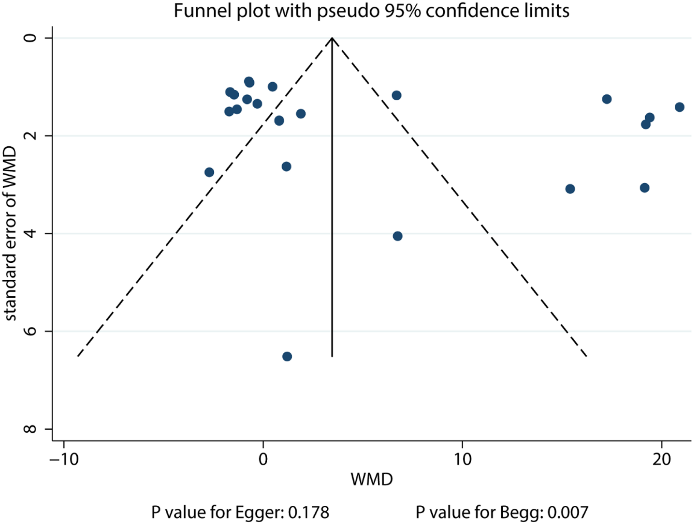


**Figure S1.** Funnel plot for BCVA.


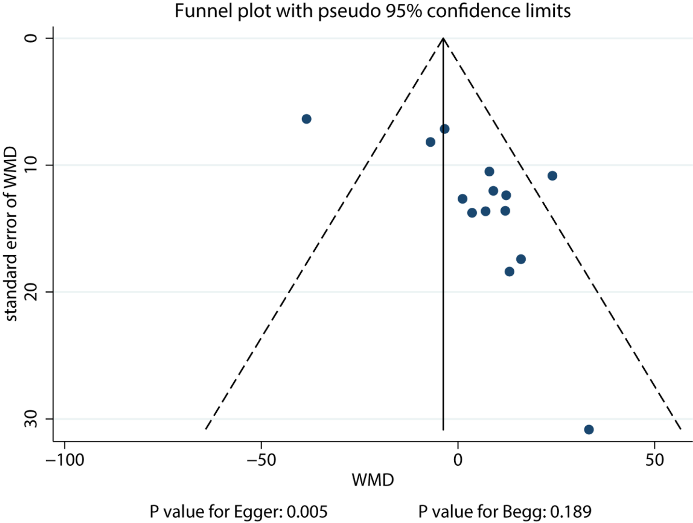


**Figure S2.** Funnel plot for central macular thickness.


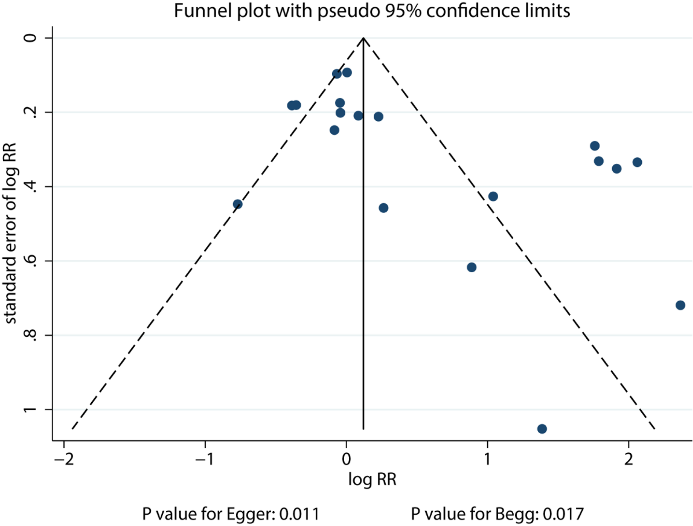


**Figure S3.** Funnel plot for gain of 15 or more letters visual acuity.


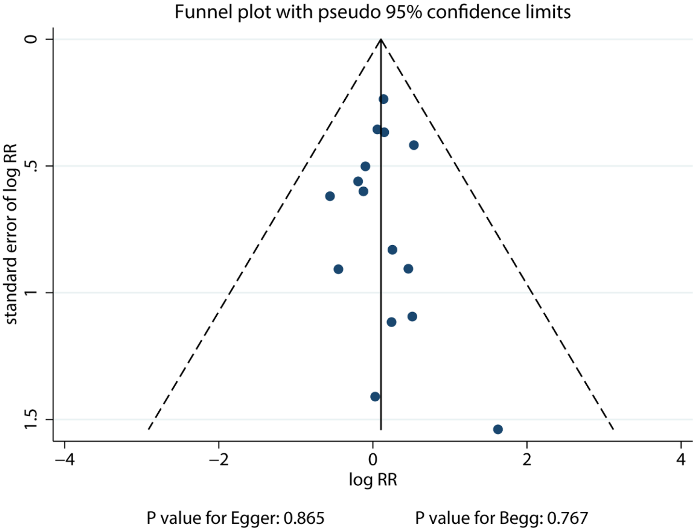


**Figure S4.** Funnel plot for death.


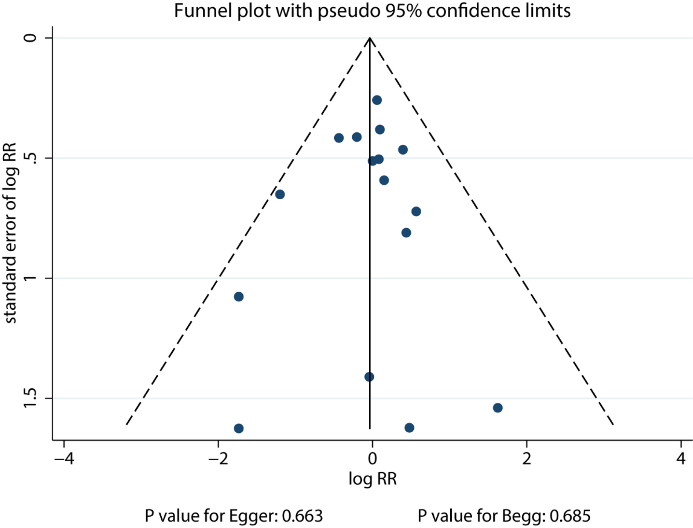


**Figure S5.** Funnel plot for arteriothrombotic events.
